# Could Guillain–Barré syndrome be triggered by COVID‐19 vaccination?

**DOI:** 10.1002/ccr3.5237

**Published:** 2022-01-18

**Authors:** Maya Aldeeb, Lina Okar, Safa S. Mahmud, Gholam A. Adeli

**Affiliations:** ^1^ Department of Medical Education Hamad Medical Corporation Doha Qatar; ^2^ Department of Neurology Movement Disorder and Neurophysiology Hamad Medical Corporation Doha Qatar

**Keywords:** corona virus, COVID‐19, Guillain–Barré syndrome, polyradiculopathy, vaccine

## Abstract

Due to SARS‐COV‐2 (COVID‐19) pandemic and its catastrophic impact on society, the FDA granted emergency use authorization for some vaccines. Possible rare side effects could not have been observed in this relatively short period. We are reporting an elderly lady with multiple comorbidities who presented with progressive lower limb weakness that started seven days after receiving the first dose of the COVID‐19 vaccine. The electrodiagnostic study showed demyelinating polyneuropathy with secondary axonal degeneration consistent with Guillain–Barré syndrome. We ruled out other possible causes for GBS, suggesting a postvaccine nature for her presentation. The patient received intravenous immunoglobulin (IVIG) for five days and gradually improved, which supports our initial diagnosis.

## Graphical abstract

As vaccination campaigns are being held worldwide, reporting incidental findings postvaccination is crucial for public awareness and establishing vaccine safety. Statistically, the risk of not taking any of the approved COVID‐19 vaccines greatly outweighs the risk of developing rare side effects.

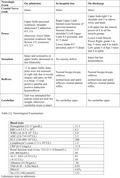

## 1 INTRODUCTION

Widespread vaccination for COVID‐19 is essential to control the current pandemic. To date, the development of new vaccines has been a long process that typically takes anywhere from 10 to 15 years.^1^ Nonetheless, in public health crises, such as the COVID‐19, exceptional measures were needed to expedite the availability and use of remedies, such as vaccines.^2^ On December 11, 2020, the FDA released an emergency use authorization (EUA) for the Pfizer‐BioNTech COVID‐19 (BNT162b2) vaccine, which is an mRNA vaccine encoding the prefusion spike glycoprotein of SARS‐CoV‐2.^3^ When we reported this case, this vaccine had not been fully approved or licensed by the FDA or the Qatar Ministry of Public Health.

With the pervasion of COVID‐19 outbreak, many studies proposed the possible neurological effects of this novel virus, for example, the spectrum of Guillain–Barré syndrome (GBS) after SARS‐CoV‐2. Furthermore, the link between GBS spectrum and many viral vaccines was hypothesized in previous evidence.^4^

As Pfizer‐BioNTech COVID vaccine is still not fully approved by the FDA, it is important to report any possible side effects. In this report, we are highlighting the fact that our patient, an 81‐year‐old lady, had GBS one week after receiving the first dose of Pfizer‐BioNTech COVID‐19 vaccine. This sequence proposed a possible correlation between BNT162b2 vaccine and GBS.

## 2 CASE PRESENTATION

An 81‐year‐old woman presented to the emergency department on January 11, 2021, with a complaint of generalized weakness for 7 days. She had a history of hypertension, type II diabetes mellitus, dyslipidemia, uncomplicated left knee arthroplasty one year back, and right knee arthroplasty 20 days before admission. She received the first dose of COVID‐19 vaccine on December 28, 2020, and described feeling fatigued and not in her usual state since the vaccination. After seven days, weakness was progressive and started in the lower limbs that then moved to the upper limbs. She also had paresthesia in the lower limbs up to the knees, and the tingling sensation in the hands and feet was different from her usual sensory complaints in the feet related to DM. There was no history of fever, URTI symptoms, or GI symptoms.

On admission, vitals showed blood pressure of 120/77 mm Hg, respiratory rate of 19 breaths per minute, oxygen saturation of 99%, and oral temperature of 36.6°C. She was oriented to person, place, and time. The patient was admitted as a suspected GBS case. Neurological examination on admission, during hospital stay, and upon discharge is summarized in Table 1, and the rest of the physical examination was unremarkable. Laboratory results are illustrated in Table 2.

**TABLE 1:** Neurological examination

Neurologic Exam	On admission	In‐hospital stay	On discharge
Cranial nerve examination	Intact	Intact	Intact
Power	Upper limbs proximal weakness: shoulder abduction3/5 adduction 4/5, 5/5 Otherwise, lower limbs proximal weakness: hip flexion 3/5, extension 4/5, 5/5	Right Upper Limb – limited exam because of previous humerus fracture (frozen shoulder?) left upper limb 4/5 proximal, and 4+/5 distal Lower limbs 4/5 proximal, and 4+/5 distal	Upper limb right 3 in shoulder and 3 in elbow, wrist, and hand Left upper has the muscle power of 4 in all the muscle groups Lower limb muscle power right: grade 3 in hip, 3+knee and 4 in ankle Left: grade 3 in hip, 3 knee, and 4 in ankle
Sensation	Intact and symmetric in upper limbs, bilaterally decreased in feet	No sensory deficit	Intact but has paresthesia
Reflexes	+2 in upper limbs and knee jerks were not assessed in right side due to recent surgery and pain, on left it is brisk +3 with positive patellar and positive adduction hyperreflexia	Normal biceps/triceps reflexes Normal knee and ankle reflexes and normal plantar reflex	Normal biceps/triceps reflexes Normal knee and ankle reflexes and normal plantar reflex
Cerebellar	Gait was attempted, but patient could not bear her weight, and otherwise, cerebellar examination is intact	No cerebellar signs	No cerebellar signs

**TABLE 2:** Laboratory tests on admission

Blood tests	
Hemoglobin (13–17 gm/dl)	11.3
RBCs (4.5–5.5 × 10^6^)	4.2
WBCs (4–10 × 10^3^/UL)	5.0
ANC (2.0–7.0 × 10^3^/UL)	3.2
PLT (150–400 × 10^3^/UL)	260
Lymphocyte count (1–3 × 10^3^/UL)	1.2
CRP (0–5 Umg/L)	11.9
Renal function tests (urea/Cr) (2.1– 8.8mmol/L) (44–80 Umoll/L)	5.5 56
ALT/AST (0–33 U/L) (0–32 U/L)	81 10
Albumin (35–50 gm/L)	36
TSH (0.30–4.20) mIU/mL	0.91
HBA1C	6.8%
Na (133–146) mmol/L	133

Lumbar puncture was attempted but failed. It was challenging due to the vague landmarks, so we tried through two tunnels with thin then thick pink needles, but it was a dry tap.

Nerve conduction study (NCS) and electromyography were performed and in the bilateral median motor nerves showed the following: prolonged distal motor latencies (DMLs), reduced compound muscle action potential (CMAP) amplitude with slight proximal temporal dispersion, and borderline conduction velocities (CVs) along with prolonged F‐wave latencies. Bilateral ulnar motor nerves showed normal DML, normal CMAP amplitude, but borderline CV along with absent F wave. Bilateral tibial and peroneal nerves showed significant temporal dispersion, low CMAP amplitude, and slow CVs along with prolonged nonsignificant F waves. Bilateral ulnar sensory nerves showed prolonged onset latencies, slow conduction velocities, but normal sensory nerve action potential (SNAP) amplitude. Left median sensory showed no response (mixed palmer and digit 2). Right median sensory showed prolonged latency and slow CVs (mixed palmar and digit 2). Right sural nerve showed reduced SNAP amplitude. Left sural sensory showed no response. The reflex of bilateral tibial and median showed absent response.

The abnormal electrodiagnostic study gave evidence of demyelinating polyneuropathy with secondary axonal degeneration consistent with acute sensory and motor polyradiculoneuropathy (Guillain–Barré syndrome).

On follow‐up examination, power deteriorated in the lower limbs and reached 3/5, so after the result of NCS and making sure that IgA level is normal, IVIG was started for a total of 5 days and the patient reported improvement after 3 days. The patient was transferred to a rehabilitation center for physiotherapy and then discharged after 24 days with remarkable improvement in power and independence level. After two weeks of discharge, the patient was almost back to her functional status in the follow‐up appointment.

## 3 DISCUSSION

According to the World Health Organization, COVID‐19 has affected more than 50 million people worldwide.^5^ Public health tackled this sudden, intolerable burden with new humanitarian settings and restrictions, changing the lives of many people. International efforts gathered to beat this pandemic by methods of prevention, such as vaccination trials. More than 120 COVID‐19 vaccine candidates were in the process within the first 5 months of 2020.^1^

Commercial vaccine industrialists and other entities have been using different technologies including inactivated virus, small‐activating RNA, mRNA, DNA, protein, and nonreplicating viral vector aiming to develop the most effective and safe COVID‐19 vaccine.^1^, ^3^ Despite the scarce data on COVID‐19 vaccines, the FDA issued an emergency use authorization (EUA) for two vaccines, Moderna COVID‐19 vaccine and Pfizer‐BioNTech COVID‐19 vaccine.^2^

Pfizer/BioNTech COVID‐19 vaccine is an mRNA vaccine that uses genetically engineered mRNA, which carries the foreign gene for the whole S protein. Although approval of Pfizer‐BioNTech Vaccine varies worldwide,^3^ it has been approved for emergency use by the Department of Pharmacy and Pharmaceutical Control in the Ministry of Public Health in Qatar. A vaccination campaign was started with the high‐risk population such as diabetic patients, intending to protect the most vulnerable groups.

The most common side effects reported in phase 3 of the vaccine study were mild‐to‐moderate pain at the injection site, fatigue, and headache. Most people experienced them after the second dose. Nevertheless, the study was not large enough to reliably reveal less common adverse events.^3^, ^6^

It is known that pathogens trigger an immune response. However, as COVID‐19 is still a novel emerging pathogen, the immunological changes are still vague. Few changes in the immune system were described in the literature, for example, abnormality in cytokine/chemokine production, hyperactivity of T cells and increased activated monocytes, neutrophils, and macrophages. Thus, COVID‐19 may mimic the inflammatory response in autoinflammatory and autoimmune diseases.^5^, ^7^

Neurological manifestations were described in one‐third of patients with COVID‐19. Some of these neurological symptoms have proved to be quite specific, for example, ageusia and insomnia. Some evidence proposed a correlation between GBS and COVID‐19.^8^

Guillain–Barré syndrome is an acute inflammatory demyelinating polyradiculoneuropathy that causes muscle weakness and paralysis, resulting in respiratory failure. GBS is one of the leading grounds of flaccid paralysis, with an annual incidence of 1.7 persons per 1,000,000 population. Half of GBS patients have an antecedent infection, but the exact etiology behind GBS is yet unknown. Still, in most of the cases, preceding infection is identified, like Campylobacter jejuni, Zika virus, human immunodeficiency virus (HIV), cytomegalovirus, Epstein–Barr virus, influenza virus, enteroviruses, herpes simplex virus, Mycoplasma pneumonia, and Haemophilus influenzae. However, the link between COVID‐19 infection and GBS is not yet confirmed.^8^ Nonetheless, when it comes to vaccination, a temporality association between GBS and many vaccines was hinted or established, like the swine flu vaccine in 1976, which increased the number of GBS cases in the six weeks after vaccination.^6^ Other vaccinations correlated to GBS are hepatitis B, measles/mumps/rubella (MMR), human papillomavirus (HPV), meningococcal quadrivalent, influenza, and rabies vaccines. Although there is no proven relationship between the COVID‐19 vaccine and GBS, reporting any suspected case is essential, as we need larger vaccine studies to reliably detect less common adverse events.^6^

On the other hand, GBS might be a sequel to a surgical procedure. Two studies mentioned that the median duration between the procedure and the onset of the first GBS symptoms was between 16 and 19 days.^7^ The primary reported surgeries were gastrointestinal, orthopedic, neurologic, cardiac, and gynecologic surgeries.

It is worth mentioning that our patient had a knee arthroplasty 20 days prior to developing the ascending weakness. At that time, the patient was discharged without complications 3 days after surgery.

Our patient is an elderly lady who was diagnosed with GBS after presenting with ascending muscle weakness 7 days after the first dose of Pfizer‐BioNTech COVID‐19 vaccine and 20 days after arthroplasty.

## 4 CONCLUSIONS

The urgent need for vaccines led to fast‐tracking vaccine development and authorizing emergency use by the FDA for some vaccines. Since the safety studies were not extensive enough to discover rare side effects of the vaccines, it is crucial that physicians report suspected adverse events. In this case report, we tried to highlight a clear temporal association between GBS and COVID‐19 vaccine. We must keep a high index of suspicion for patients with new‐onset GBS in the COVID‐19 era, especially after vaccination. The clinical implications of this association warrant further research.

To conclude, risks of adverse events, such as GBS, cannot be counted as a compelling cause to avoid the administration of currently recommended vaccines, but it is vital to report and gather all the evidence about the new vaccines to achieve the best safety profile.

## CONFLICT OF INTEREST

The authors report no conflicts of interest.

## AUTHORS CONTRIBUTIONS

All authors equally contributed to writing the manuscript.

## ETHICAL APPROVAL

Consent was obtained from the patients.

## CONSENT

Written informed consent was obtained from the patient to publish this report in accordance with the journal's patient consent policy.

## Supporting information

Supplementary Material

## Data Availability

All data related to this article are available upon request.
