# Supplementary material for: Could Guillain–Barré syndrome be triggered by COVID‐19 vaccination?
Source: Clin Case Rep. 2022 Jan 18;10(1):e05237. doi: 10.1002/ccr3.5237 (PMC8765088; doi:10.1002/ccr3.5237)

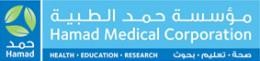


HAMAD MEDICAL CORPORATION

Neurophysiology Lab Hamad General Hospital NCS & EMG Report

Patient Information

Generated:

13/01/2021

| ID |  | Date of birth |  |
| --- | --- | --- | --- |
| Name |  | In Out | In |
| Sex | Female | Doctor | DR GHOLAM |
| Age |  | Examiner |  |
| Weight |  | Referring Department | (Unknown) |
| Height |  | Examination Date | 13/01/2021 |
| History | LOWER LIMBS WEAKNES POST COVID VACIN | | |
| Comment |  | | |

Remarks

NCS / EMG findings: Bilateral median motor nerves showed prolonged distal motor

latencies, reduced CMAP amplitude with slight proximal temporal dispersion and borderline conduction velocities along with prolonged F wave latencies. Bilateral ulnar motor nerves showed normal DML, normal CMAPs amplitude but borderline CVs along with absent F waves. Bilateral tibial and peroneal nerves showed significant temporal dispersion, low CMAPs amplitude, slow CVs along with prolonged non-significant F waves. Bilateral ulnar sensory nerves showed prolonged onset latencies, slow conduction velocities but normal SNAPs amplitude.

left median sensory showed no response (mixed palmar and digit 2). right median sensory showed prolonged latency, slow CVs (mixed palmar and digit2). Right sural nerve showed reduced SNAPs amplitude. Left Sural sensory showed no response. H reflex of bilateral tibial and median showed absent response.

Conclusion:

This is an abnormal electrodiagnostic study, there is electrophysiological evidence of demyelinating polyneuropathy with secondary axonal consistent with acute sensory and motor polyneuroradiculopathy **(Guillain Baree Syndrome GBS)**

Signature:

Table (A): Motor Nerve Conduction Study

| Site | Lat. (ms) | N.D. | Dur. (ms) | Amp. | N.D. | Area | Stim. (mA) | Segment | Dist. (mm) | Intvl. (ms) | NCV  (m/s) | CCV  (m/s) | N.D. | Temp |
| --- | --- | --- | --- | --- | --- | --- | --- | --- | --- | --- | --- | --- | --- | --- |

Median Left Temperature:

| Wrist | 8.5 |  | 9.2 | 2.9mV |  | 14.1mVms | 23.8 | *Wrist |  | 8.5 |  |  |  | 21.4 |
| --- | --- | --- | --- | --- | --- | --- | --- | --- | --- | --- | --- | --- | --- | --- |
| Elbow | 12.8 |  | 9.6 | 2.3mV |  | 10.7mVms | 23.8 | Wrist-Elbow | 210 | 4.3 | 48.8 |  |  | 21.5 |

Median Right Temperature:

| Wrist | 6.5 |  | 7.7 | 2.8mV |  | 9.8mVms | 20.2 | *Wrist |  | 6.5 |  |  |  | 21.3 |
| --- | --- | --- | --- | --- | --- | --- | --- | --- | --- | --- | --- | --- | --- | --- |
| Elbow | 11.5 |  | 8.5 | 2.8mV |  | 11.4mVms | 20.2 | Wrist-Elbow | 220 | 5.0 | 44.0 |  |  | 21.3 |
|  |  |  |  |  |  |  |  | Elbow-Axilla |  |  |  |  |  |  |

Ulnar Left Temperature:

| Wrist | 5.6 |  | 7.4 | 5.0mV |  | 18.6mVms | 23.2 | *Wrist |  | 5.6 |  |  |  | 21.5 |
| --- | --- | --- | --- | --- | --- | --- | --- | --- | --- | --- | --- | --- | --- | --- |
| D.elbow | 9.9 |  | 7.7 | 4.4mV |  | 16.6mVms | 28.2 | Wrist-D.elbow | 210 | 4.3 | 48.8 |  |  | 21.5 |
| P.elbow | 12.0 |  | 7.4 | 3.1mV |  | 12.2mVms | 39.2 | D.elbow-P.elbow | 70 | 2.1 | 33.3 |  |  | 21.5 |

Ulnar Right Temperature:

| Wrist | 4.2 |  | 6.9 | 6.2mV |  | 20.6mVms | 29.2 | *Wrist |  | 4.2 |  |  |  | 21.3 |
| --- | --- | --- | --- | --- | --- | --- | --- | --- | --- | --- | --- | --- | --- | --- |
| Elbow | 9.6 |  | 7.6 | 5.5mV |  | 22.3mVms | 47.2 | Wrist-Elbow | 230 | 5.4 | 42.6 |  |  | 21.2 |
| Axilla | 11.3 |  | 6.9 | 5.4mV |  | 21.2mVms | 47.2 | Elbow-Axilla | 80 | 1.7 | 48.5 |  |  | 21.2 |

Peroneal Left Temperature:

| Ankle | 8.7 |  | 18.9 | 1.0mV |  | 4.2mVms | 43.4 | *Ankle |  | 8.7 |  |  |  | 21.2 |
| --- | --- | --- | --- | --- | --- | --- | --- | --- | --- | --- | --- | --- | --- | --- |
| Head of fibula | 17.0 |  | 21.1 | 0.4mV |  | 3.2mVms | 43.4 | Ankle-Head of fibula | 310 | 8.3 | 37.3 |  |  | 21.2 |
| Popliteal | 19.8 |  | 17.6 | 0.4mV |  | 2.7mVms | 43.4 | Head of fibula-Popliteal | 80 | 2.8 | 28.6 |  |  | 21.2 |

Peroneal Right Temperature:

| Ankle | 7.4 |  | 8.4 | 2.0mV |  | 6.9mVms | 30.2 | *Ankle |  | 7.4 |  |  |  | 21.2 |
| --- | --- | --- | --- | --- | --- | --- | --- | --- | --- | --- | --- | --- | --- | --- |
| Head of fibula | 15.6 |  | 9.4 | 1.5mV |  | 7.2mVms | 57.0 | Ankle-Head of fibula | 300 | 8.3 | 36.4 |  |  | 21.2 |
| Popliteal | 17.9 |  | 10.4 | 0.7mV |  | 4.5mVms | 65.6 | Head of fibula-Popliteal | 75 | 2.3 | 33.3 |  |  | 21.2 |

Tibial Left Temperature:

| Ankle | 7.4 |  |  | 418.7uV |  |  | 52.4 | *Ankle |  | 7.4 |  |  |  | 21.2 |
| --- | --- | --- | --- | --- | --- | --- | --- | --- | --- | --- | --- | --- | --- | --- |
| Popliteal | 17.5 |  | 20.8 | 360.3uV |  | 2558.6uV  ms | 57.0 | Ankle-Popliteal | 360 | 10.1 | 35.6 |  |  | 21.2 |

Tibial Right Temperature:

| Ankle | 8.3 |  | 9.0 | 0.6mV |  | 2.6mVms | 40.2 | *Ankle |  | 8.3 |  |  |  | 21.0 |
| --- | --- | --- | --- | --- | --- | --- | --- | --- | --- | --- | --- | --- | --- | --- |
| Popliteal | 16.8 |  | 14.3 | 0.7mV |  | 3.2mVms | 50.8 | Ankle-Popliteal | 370 | 8.5 | 43.8 |  |  | 21.0 |

Table (B): Sensory Nerve Conduction Study

| Site | Lat.1 (ms) | N.D. | Lat.2 (ms) | Amp. | N.D. | Area | Stim. (mA) | Segment | Dist. (mm) | Intvl. (ms) | NCV  (m/s) | CCV  (m/s) | N.D. | Temp |
| --- | --- | --- | --- | --- | --- | --- | --- | --- | --- | --- | --- | --- | --- | --- |

Median Left Temperature:

| Wrist |  |  |  |  |  |  | 21.2 | Wrist |  |  |  |  |  | 21.5 |
| --- | --- | --- | --- | --- | --- | --- | --- | --- | --- | --- | --- | --- | --- | --- |
| Elbow |  |  |  |  |  |  | 21.2 | Wrist-Elbow |  |  |  |  |  | 21.5 |
|  |  |  |  |  |  |  |  | Elbow-Axilla |  |  |  |  |  |  |

Median Right Temperature:

| Wrist | 4.1 |  | 5.4 | 9.2uV |  | 0.5uVms | 12.2 | Wrist | 140 | 4.1 | 33.8 |  |  | 21.3 |
| --- | --- | --- | --- | --- | --- | --- | --- | --- | --- | --- | --- | --- | --- | --- |
| Palm | 2.3 |  | 3.3 | 9.5uV |  | 0.7uVms | 16.8 | Palm | 80 | 2.3 | 34.2 |  |  | 21.2 |
|  |  |  |  |  |  |  |  | Palm-Axilla |  |  |  |  |  |  |

Ulnar Left Temperature:

| Wrist | 2.6 |  | 3.8 | 20.0uV |  | 1.6uVms | 19.6 | Wrist | 100 | 2.6 | 38.2 |  |  | 21.5 |
| --- | --- | --- | --- | --- | --- | --- | --- | --- | --- | --- | --- | --- | --- | --- |
|  |  |  |  |  |  |  |  | Wrist-Elbow |  |  |  |  |  |  |
|  |  |  |  |  |  |  |  | Elbow-Axilla |  |  |  |  |  |  |

Ulnar Right Temperature:

| Wrist | 2.5 |  | 3.7 | 22.4uV |  | 3.0uVms | 8.4 | Wrist | 90 | 2.5 | 36.6 |  |  | 21.2 |
| --- | --- | --- | --- | --- | --- | --- | --- | --- | --- | --- | --- | --- | --- | --- |
|  |  |  |  |  |  |  |  | Wrist-Elbow |  |  |  |  |  |  |
|  |  |  |  |  |  |  |  | Elbow-Axilla |  |  |  |  |  |  |

Sural Left Temperature:

| Sural |  |  |  |  |  |  | 24.6 | Sural |  |  |  |  |  | 21.2 |
| --- | --- | --- | --- | --- | --- | --- | --- | --- | --- | --- | --- | --- | --- | --- |
|  | 2.9 |  | 3.9 | 5.9uV |  | 0.3uVms | 21.4 |  |  |  |  |  |  |  |

Sural Right Temperature:

| Sural | 3.1 |  | 4.4 | 4.1uV |  | 0.7uVms | 22.2 | Sural |  | 3.1 |  |  |  | 21.3 |
| --- | --- | --- | --- | --- | --- | --- | --- | --- | --- | --- | --- | --- | --- | --- |


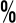

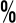

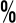

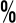
Table (C): F-wave

| Nerve | Side | Stim.Site | F-Lat. | F-Lat.N.D. | M Lat. | F-M Lat. | F-Occurr. | Distance | FWCV | N.D. |
| --- | --- | --- | --- | --- | --- | --- | --- | --- | --- | --- |
| Median | Left | Wrist | 39.8ms |  | 0.0ms | 39.8ms | 9 / 9 , 10  0 |  | *** |  |
| Median | Right | Wrist | 36.5ms |  | 0.0ms | 36.5ms | 5 / 10 , 50 |  | *** |  |
| Ulnar | Left | Wrist | 75.0ms |  | 0.0ms | 75.0ms | 3 / 9 , 33 |  | *** |  |
| Ulnar | Right | Wrist | 34.5ms |  | 20.0ms | 14.5ms | 8 / 10 , 80 |  | *** |  |
| Peroneal | Left | Ankle | *** |  | 0.0ms | *** | 0 / 11 , 0 |  | *** |  |
| Peroneal | Right | Ankle | 62.2ms |  | 0.0ms | 62.2ms | 3 / 12 , 25 |  | *** |  |
| Tibial | Left | Ankle | 67.4ms |  | 39.9ms | 27.5ms | 3 / 11 , 27 |  | *** |  |
| Tibial | Right | Ankle | 68.9ms |  | 0.0ms | 68.9ms | 4 / 13 , 30 |  | *** |  |


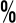

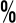

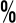

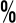
Table (D): H-reflex

| Nerve | Median | Side | Left |  | |
| --- | --- | --- | --- | --- | --- |
| Stim.Site |  | Rec.Site |  |  |  |
| M-Latency | 3.2ms | M-Amp.Max | 0.4mV |  |  |
| H-Latency |  | H-Amp.Max |  | H/M.Ratio |  |

| Nerve | Tibial | Side | Left |  | |
| --- | --- | --- | --- | --- | --- |
| Stim.Site |  | Rec.Site |  |  |  |
| M-Latency |  | M-Amp.Max |  |  |  |
| H-Latency |  | H-Amp.Max |  | H/M.Ratio |  |

| Nerve | Tibial | Side | Right |  | |
| --- | --- | --- | --- | --- | --- |
| Stim.Site |  | Rec.Site |  |  |  |
| M-Latency | 4.9ms | M-Amp.Max | 2.7mV |  |  |
| H-Latency |  | H-Amp.Max |  | H/M.Ratio |  |


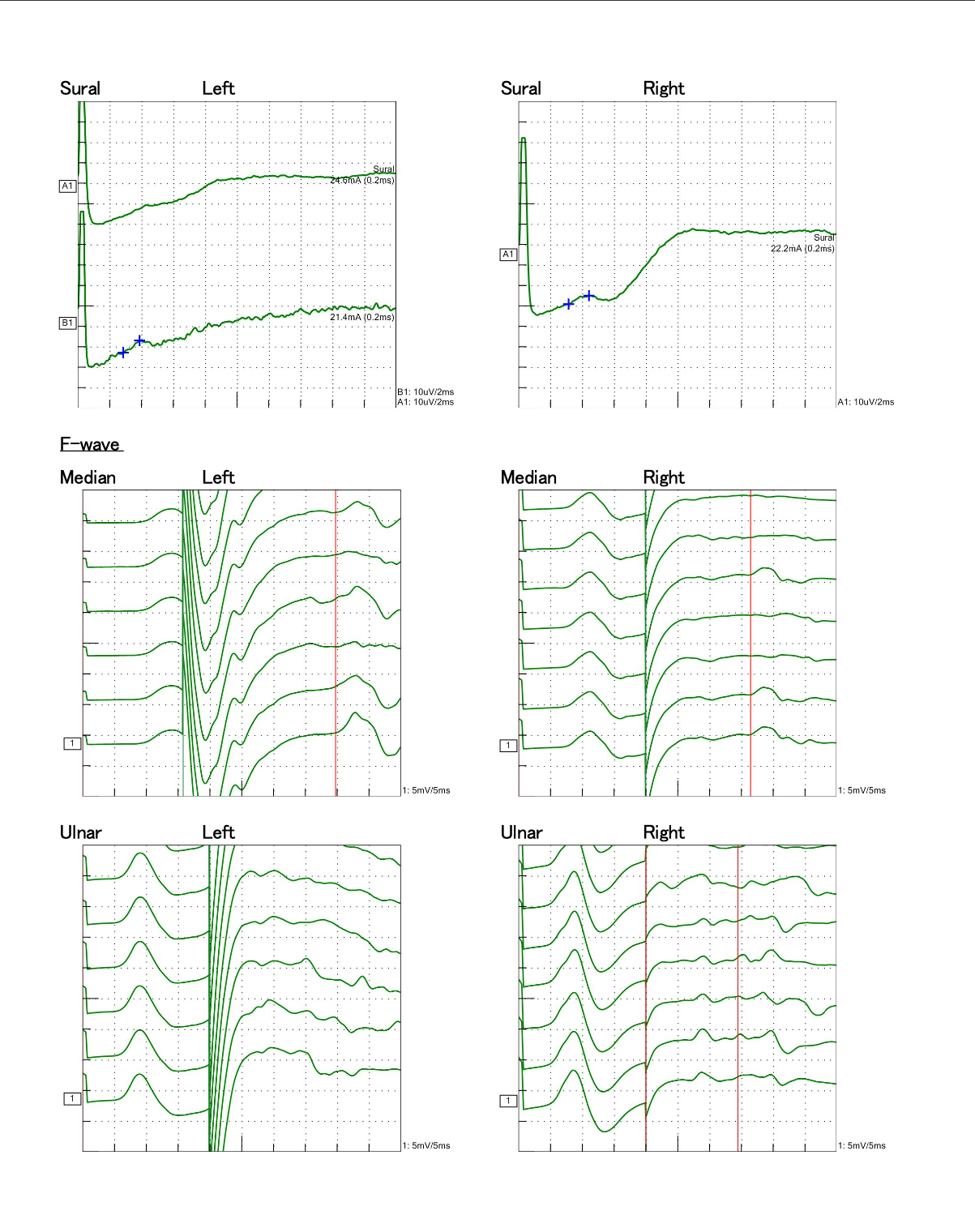

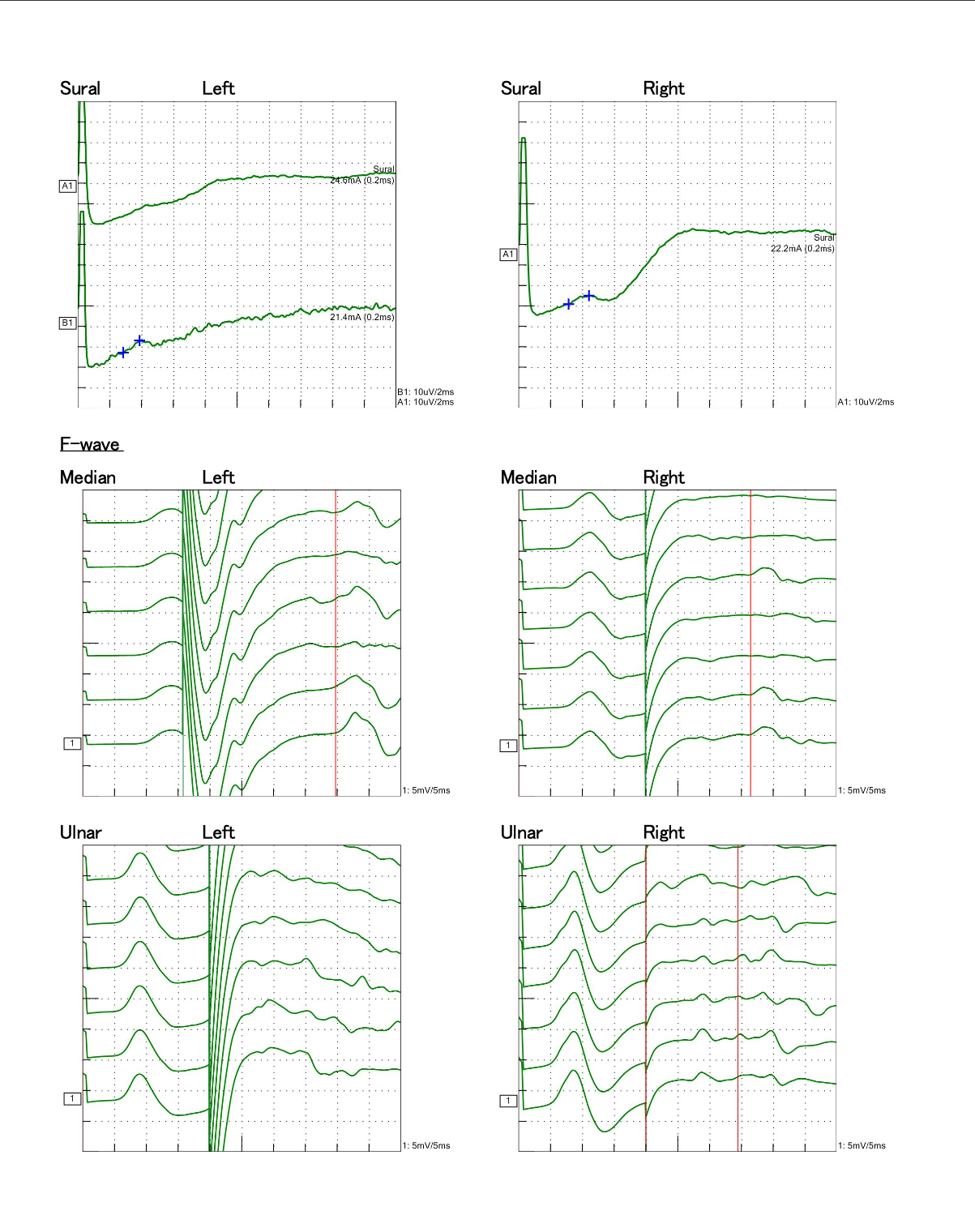

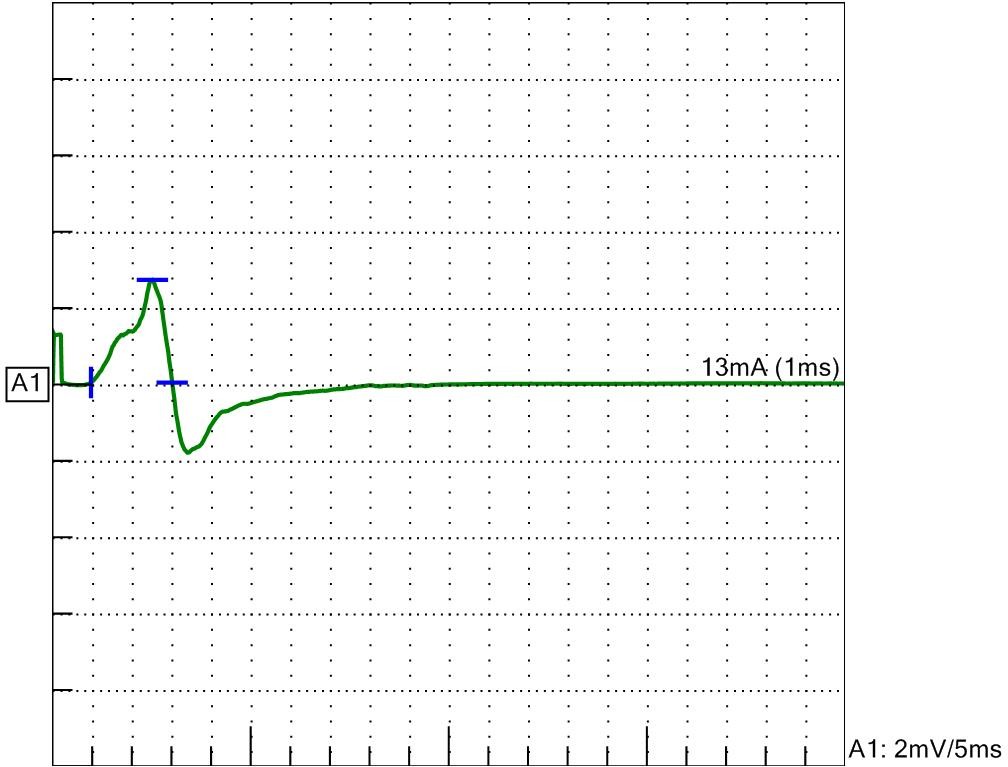

Supplement: Supplementary file 1 — Supplementary Material [file CCR3-10-e05237-s001.docx]
